# Hydrogen Sulfide Prevents Elastin Loss and Attenuates Calcification Induced by High Glucose in Smooth Muscle Cells through Suppression of Stat3/Cathepsin S Signaling Pathway

**DOI:** 10.3390/ijms20174202

**Published:** 2019-08-27

**Authors:** Ye-Bo Zhou, Hong Zhou, Li Li, Ying Kang, Xu Cao, Zhi-Yuan Wu, Lei Ding, Gautam Sethi, Jin-Song Bian

**Affiliations:** 1 Department of Pharmacology, Yong Loo Lin School of Medicine, National University of Singapore, Singapore 117597, Singapore; 2 Department of Physiology, Nanjing Medical University, Nanjing 211166, China; 3 Department of Pathophysiology, Xuzhou Medical University, Xuzhou 221004, China; 4 National University of Singapore (Suzhou) Research Institute (NUSRI), Suzhou Industrial Park, Suzhou 215123, China

**Keywords:** hydrogen sulfide, calcification, smooth muscle cells, Stat3, cathepsin S, elastin

## Abstract

Vascular calcification can be enhanced by hyperglycemia. Elastin loss in tunica media promotes the osteogenic transformation of smooth muscle cells (SMCs) and involves arterial medial calcification (AMC) that is associated with a high incidence of cardiovascular risk in patients with type 2 diabetes. Here, we tested whether hydrogen sulfide (H_2_S), an endogenous gaseous mediator, can prevent elastin loss and attenuate calcification induced by high glucose in SMCs. Calcification was induced by high glucose (4500 mg/L) in human aortic SMCs (HASMCs) under the condition of calcifying medium containing 10 mM β-glycerophosphate (β-GP). The experiments showed that NaHS (an H_2_S donor, 100 μM) mitigated the calcification of HASMCs treated with high glucose by decreasing calcium and phosphorus levels, calcium deposition and ALP activity and inhibited osteogenic transformation by increasing SMα-actin and SM22α, two phenotypic markers of smooth muscle cells, and decreasing core binding factor α-1 (Cbfα-1), a key factor in bone formation, protein expressions in HASMCs. Moreover, NaHS administration inhibited the activation of Stat3, cathepsin S (CAS) activity and its expression, but increased the level of elastin protein. Pharmacological inhibition or gene silencing Stat3 not only reversed elastin loss, but also attenuated CAS expression. Inhibition of CAS alleviated, while CAS overexpression exacerbated, elastin loss. Interestingly, overexpression of wild type (WT)-Stat3, but not its mutant C259S, elevated CAS protein expression and reduced elastin level. Moreover, NaHS induced S-sulfhydration in WT, but not in the C259S Stat3. These data suggest that H_2_S may directly regulate Cys259 residue in Stat3 and then impair its signaling function. Our data indicate that H_2_S may attenuate vascular calcification by upregulating elastin level through the inhibition of Stat3/CAS signaling.

## 1. Introduction

Vascular calcification (VC) is considered to be an active, regulative process similar to osteogenesis [1]. Arterial medial calcification (AMC) contributes directly to cardiovascular morbidity and mortality in elderly individuals and patients with diabetes mellitus (DM) or chronic kidney disease (CKD) [1,2]. High glucose or a disordered mineral metabolism (e.g., elevated phosphorus level) may induce osteogenic differentiation of vascular smooth muscle cells (VSMCs) [3,4], which may further contribute to the substantial increase of cardiovascular events (CVE) [5,6]. AMC could be driven by the dysmetabolic milieus of diabetes and uremia such as hyperglycemia and hyperphosphatemia [3]. VC often begins early and gets severe during the course of CKD, particularly in individuals with DM. This explains why the prevalence, extent, and progression of VC as well as the incidence of CVE are higher in diabetics with concomitant CKD [7,8,9].

The physiological and pathophysiological importance of hydrogen sulfide (H_2_S) has been widely explored in the cardiovascular system [10,11,12]. The production of H_2_S in arterial wall is mainly catalyzed by cystathionine-γ-lyase (CSE) [13], and blunted CSE activity is associated with hypertension and myocardial dysfunction in diabetes [14]. Moreover, CSE/H_2_S system is essential for the maintenance of VSMCs differentiation. Exogenous application of H_2_S inhibits VSMCs proliferation in hyperglycemic state [15]. In diabetic rats, it not only decreases blood glucose, but also plays beneficial roles in cardiovascular diseases like atherosclerosis and diabetic cardiomyopathy [14,16,17,18]. Although H_2_S has been shown to ameliorate phosphate-induced calcification in VSMCs, or vitamin D3 and nicotine combination-induced calcification in rats [13], whether H_2_S inhibits high glucose-induced mineralization in aortic smooth muscle cells (HASMCs) has not been studied.

VC, vascular inflammation, and endoplasmic reticulum stress are pathological processes occurring in the vessel wall under the condition of hyperglycemia [19,20]. Signal transducer and activator of transcription-3 (Stat3), one of Stat family members, can be activated by inflammation [21]. It mediates the inflammation and immune responses which contribute to signal integration in the vascular dysfunction [22,23]. Stat3 is also closely associated with VC [24] and high glucose-induced inflammation and endoplasmic reticulum stress [25].

Cathepsin S (CAS), a potent protease, cleaves elastin and generates bioactive elastin peptides, which, in turn, can incite inflammation, stimulate macrophage chemotaxis, and promote calcification [26,27]. It also co-localizes with the increased elastin breaks in the atherosclerotic plaques [28,29]. Moreover, a deficiency of CAS leads to decreased vascular inflammation, calcification and elastolytic activity in the arteries of hypercholesterolemic mice with experimental CKD [30]. Inhibition of CAS also lowers blood glucose levels in mice [31]. All of these evidence implicate that CAS induced elastolysis involves arterial calcification.

The interaction between Stat3 and CAS has been reported previously. IL-6-Stat3 controls intracellular MHC class II αβ dimer level through CAS activity in dendritic cells, and IL-10-Stat3 on MHC-II antigen presentation may occur via the inhibition of CAS expression in primary cultured human blood macrophages [32,33]. However, Stat3-mediated activation of CAS in calcification in VSMCs remains poorly understood.

Since diabetes companied by calcification is a severe disease state with increased mortality, the present study was therefore designed to clarify the protective effects of H_2_S on human aortic smooth muscle cells (HASMs) treated with a high concentration of glucose under the condition of calcifying medium containing 10 mM β-glycerophosphate (β-GP). The involvement of Stat3/CAS pathway was also studied.

## 2. Results

### 2.1. NaHS Treatment Attenuates Calcification in High Glucose (HG) Treated HASMCs.

**Figure 1:**
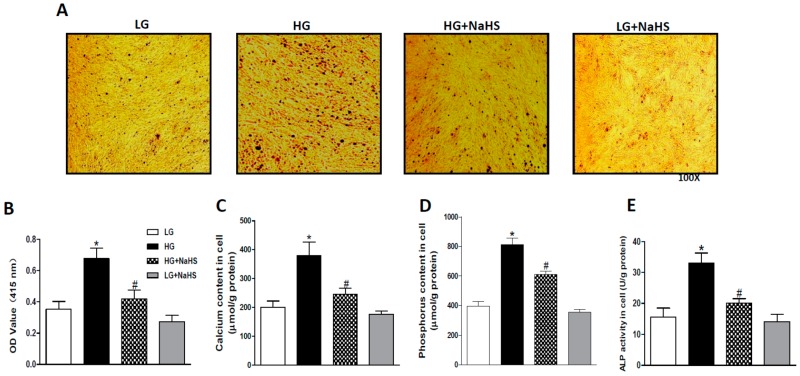
NaHS suppressed high glucose (HG)-promoted calcification in cultured human aortic smooth muscle cells (HASMCs). HASMCs were treated with LG (1000 mg/L) plus β-glycerophosphate (β-GP, 10 mM) or HG (4500 mg/L) plus β-GP for 7 or 14 days with or without NaHS (100 μM). (**A**): Alizarin red staining showing Ca deposition in cultured HASMCs 14 days after treatment with HG; (**B**,**C**): Calcium content in cultured HASMCs treated with HG for 14 days. Alizarin red was dissolved in 5% SDS and 0.5 N HCl, and the absorbance was evaluated at 415 nm to quantify the calcium content (**B**), the O-cresolphthalein colorimetric (OCPC) method was also used for measuring calcium content (**C**); (**D**): Phosphorus content in cultured HASMCs treated with HG for 14 days; (**E**): alkaline phosphatase (ALP) activity in cultured HASMCs treated with HG for 7 days. *n* = 3–5 independent experiments. Values represent the means ± SEM. * *p* < 0.05 compared to LG. # *p* < 0.05 compared to HG.

We confirmed the effect of NaHS on HG-induced calcification in HASMCs. Treatment with HG for 14 days induced obvious Ca deposition (Figure 1A), elevation of calcium (Figure 1B,C) and phosphorus (Figure 1D) levels in HASMCs. In addition, culture with HG for 7 days also caused significant increases in ALP activity (Figure 1E). All the above changes were also abolished by NaHS (100 μM) treatment.

### 2.2. NaHS Treatment Inhibits Osteogenic Transition of VSMC in HG Treated HASMCs.

**Figure 2:**
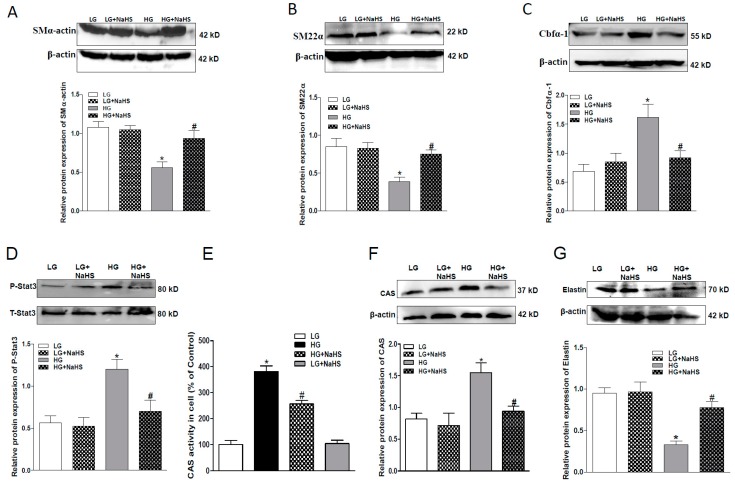
NaHS suppressed the osteogenic transition, Stat3 phosphorylation, activities and protein expression of cathepsin S (CAS), and increased the protein expression of elastin (**G**) of HG-cultured HASMCs. Western blots showing that NaHS attenuated HG-induced down-regulation in SMα-actin (**A**), SM22α (**B**), and elastin (**G**) and up-regulation of Cbfα-1 (**C**), Stat3 phosphorylation (**D**) and activities and protein expression of CAS (**E**,**F**). *n* = 3–5. Values represent the means ± SEM. * *p* < 0.05 compared to LG. ^#^ *p* < 0.05 compared to HG. The protein expressions of SMα-actin, SM22α, elastin, Cbfα-1 Stat3, CAS, and elastin were examined in HG-cultured HASMCs after NaHS (100 μM) treatment for 7 days. β-actin or total Stat3 from the same blots was used as a protein loading control.

Osteogenic transition was assessed by examination of the protein expression of SMα-actin and SM22α, two phenotypic markers of VSMC, and Cbfα-1, a key osteogenic regulator. As shown in Figure 2, the protein levels of SMα-actin and SM22α were down-regulated, whereas the protein level of Cbfα-1 was up-regulated in the HG-treated HASMCs. NaHS treatment reversed all above changes in HASMCs, thereby suggesting that H_2_S can attenuate the osteogenic transition process in smooth muscle cells. To study the signaling mechanism(s), we determined the effect of NaHS treatment on the activation of Stat3 and protein expression of CAS. As shown in Figure 2, both activities of Stat3 and CAS were elevated in HG-treated HASMCs (Figure 2D,E). Western blots demonstrated that the elevated CAS activity may be from the upregulated CAS protein expression, and NaHS treatment abolished the upregulated Stat3 activation and CAS protein expression in HG-treated HASMCs (Figure 2D,F). Consistent with previous studies, CAS plays an important role in elastin degradation and its activity regulates elastin level in aortic wall [30], we found in the present study that the application of NaHS also attenuated the reduced elastin level in HG-treated HASMCs (Figure 2G). Our findings suggest that NaHS treatment may rescue the loss of elastin through the inhibition of the activated Stat3/CAS pathway.

### 2.3. Impaired Endogenous H_2_S Generating Enzyme Activity and Expression in HG-Treated HASMCs

**Figure 3:**
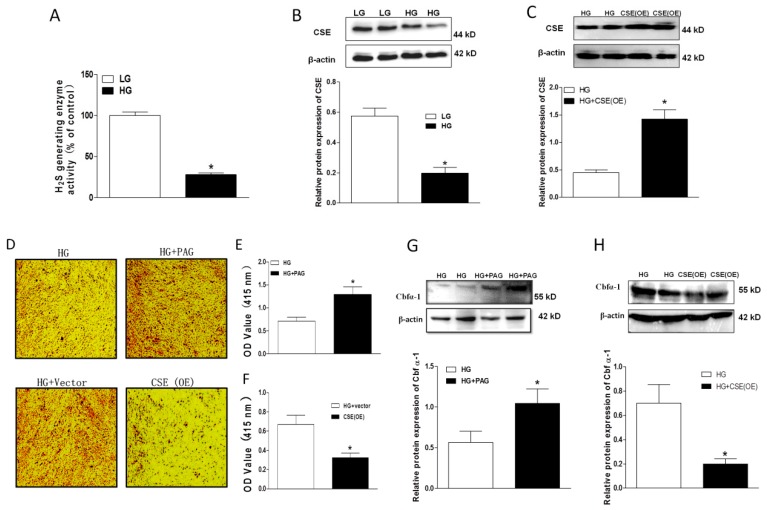
Role of endogenous H_2_S in the development of calcification. Endogenous H_2_S generating enzyme activity in HG-cultured HASMCs (**A**); Western blots showing the protein expression of cystathionine-γ-lyase (CSE) in HG-cultured HASMCs (**B**) and overexpressing CSE or using PAG (100 µM, a CSE inhibitor, 14 days) on Ca deposition (**D**–**F**) and the protein expression of Cbfα-1 (**G**,**H**). Alizan red staining and its quantitative analysis showing the effects of PAG or CSE overexpression (6 days, cells were transfected two times for obvious staining) on Ca deposition in HASMCs treated with HG (**D**–**F**); CSE protein expression for testing CSE overexpression after transfection (**C**). HG-cultured HASMCs were collected on the third day after transfection for CSE (**C**) and Cbfα-1 examination (**H**). HASMCs were treated with HG or PAG for 7 days (**G**). *n* = 3–5. Values represent the means ± SEM. * *p* < 0.05 compared to LG (**A**,**B**) or HG (**C**–**H**).

To study the role of endogenous H_2_S, we first investigated the ability of H_2_S generating enzyme in the presence of sufficient substrates (10 mM l-cystein). It was found that the H_2_S generating enzyme activity was largely reduced in HG-treated HASMCs (Figure 3A). Since CSE is the main enzyme to produce H_2_S in vascular smooth muscles, we further measured CSE protein expression in HG-treated HASMCs. Western blots demonstrated that CSE levels were also markedly downregulated in this situation (Figure 3B). These data suggest that endogenous H_2_S may be reduced during a diabetic state and the impaired endogenous H_2_S generating enzyme ability may contribute to the vascular pathology in diabetes. To clarify the role of endogenous H_2_S in calcification, we further regulated endogenous H_2_S production by either an application of PAG (a CSE inhibitor, 100 µM) to decrease endogenous H_2_S production or the overexpression (OE) of CSE to compensate the impaired endogenous H_2_S generation. Western blots demonstrated that CSE protein expression was significantly upregulated by CSE OE (Figure 3C). Alizarin red staining and calcium content data demonstrated that application of PAG induced more Ca deposition, whereas CSE OE largely reduced it (Figure 3D–F). This was consistent with their effects on the key osteogenic regulator, Cbfα-1 (Figure 3G,H).

### 2.4. Effect of Endogenous H_2_S on Stat3/CAS/Elastin Pathway

**Figure 4:**
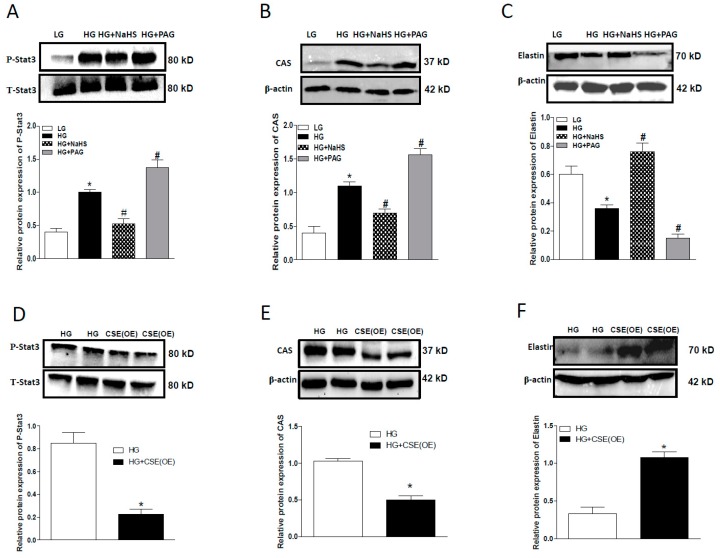
Western blots showing the effects of PAG (**A**–**C**) or CSE overexpression (**D**–**F**) on Stat3 phosphorylation (**A**,**D**), and protein expression of CAS (**B**,**E**) and elastin (**C**,**F**) in HG-cultured HASMCs after NaHS (100 μM) treatment for 7 days or CSE overexpression (**D**–**F**). HG-cultured HASMCs were collected on third day after transfection. β-actin or total Stat3 from the same blot was used as protein loading control. *n* = 3–5 experiments. Values represent the means ± SEM. * *p* < 0.05 compared to LG (**A**–**C**) or HG (**D**–**F**). ^#^ *p* < 0.05 compared to HG (**A**–**C**).

Next, we studied the role of endogenous H_2_S on Stat3/CAS/elstin pathway. Treatment of HASMCs with PAG (100 µM, for 7 days) further stimulated the Stat3/CAS pathway caused by HG (Figure 4A,B), while CSE OE decreased it (Figure 4D,E). This was further confirmed by the elastin level in HASMCs. As shown in Figure 4C,F, PAG further reduced HG-induced elastin loss (Figure 4C), whereas CSE OE upregulated it (Figure 4F).

### 2.5. CAS Is Important in Calcification of Smooth Muscle Cells

**Figure 5:**
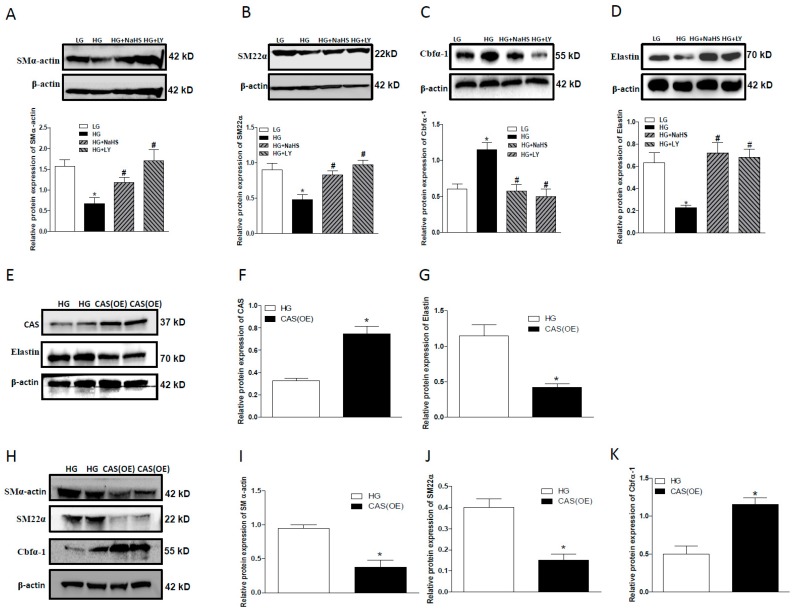
Western blots showing the effects of LY, a CAS inhibitor, or CAS overexpression on calcification and elastin expression in HG-treated HASMCs. (**A**–**D**): LY abolished the effects of HG-induced changes on SMα-actin (**A**), SM22α (B), Cbfα-1 (**C**), and elastin (D) in HASMCs treated with HG for 7 days. CAS overexpression (**E**,**F**) reduced the protein expression of elastin (**E**,**G**), SMα-actin (**H**,**I**), SM22α (**H**,**J**) and increased the protein expression of Cbfα-1 (**H**,**K**) in HASMCs treated with HG for 3 days. *n* = 3–5. Values represent the means ± SEM. * *p* < 0.05 compared to LG (**A**–**D**) or HG (**F**,**G**,**I**–**K**), ^#^ *p* < 0.05 compared to HG (**A**–**D**).

To confirm the involvement of CAS in HG-induced mineralization of HASMCs, LY3000328 (LY, a selective CAS inhibitor, 1µM) was used. Western blotting analysis showed that, similar to NaHS, LY reversed the downregulated expression of SMα-actin (Figure 5A), SM22α (Figure 5B), and elastin (Figure 5D) and upregulated level of Cbfα-1 (Figure 5C). However, the overexpression of CAS produced the opposite effects on the expression level of all these proteins (Figure 5E–K). These data indicate that CAS may promote the transformation of the osteogenic phenotype of HASMCs in a HG situation.

### 2.6. Stat3 Inhibitor or Stat3 Silencing Inhibited Osteoblastic Differentiation, Decreased CAS Expression, but Increased Elastin Expression

**Figure 6:**
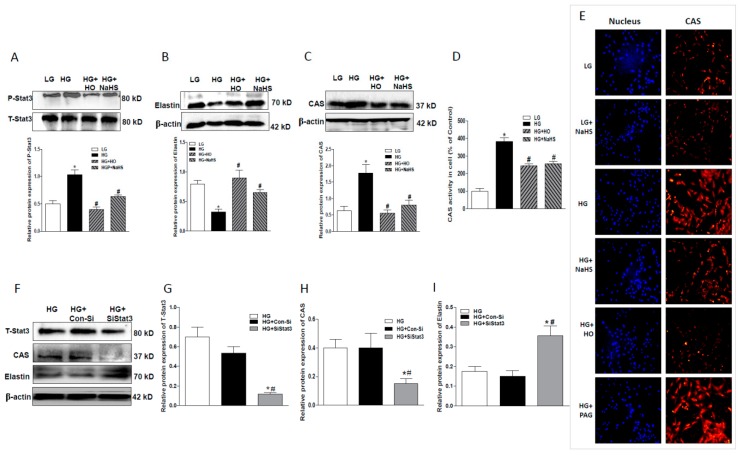
Effects of HG on Stat3 phosphorylation, CAS activity, and protein expression of elastin and CAS in HASMCs treated with HO, a Stat3 inhibitor (**A**–**D**) or upon Stat3 silencing (**F**–**I**). (**A**–**D**): HO attenuated HG-induced Stat3 phosphorylation (**A**), down-regulation of elastin protein (**B**), and up-regulation CAS protein (**C**) and CAS activity (**D**) in HASMCs treated with HG for 7 days. (**E**): Immunofluorescent staining shows that HG increased CAS protein expression and this effect was attenuated by NaHS or HO, but potentiated by PAG. CAS (red) and 4’,6-diamidino-2-phenylindole (DAPI) (blue) in HASMCs (Magnification 100×). (**F**–**I**): Stat3 SiRNA (SiStat3) (**F**,**G**) also reduced the protein expression of CAS (**H**) and elastin (**I**) in HG-treated HASMCs for 3 days. HASMCs were treated with NaHS (100 μM), HO-3867 (a Stat3 inhibitor) and PAG (a CSE inhibitor), respectively, under the condition of LG or HG for 7 days. The fluorescence images are representatives from three independent experiments. Results are from one representative experiment and relative to total Stat3 or β-actin expression. *n* = 3–5 experiments. Values represent the means ± SEM. * *p* < 0.05 compared to LG, ^#^ *p* < 0.05 compared to HG (**A**–**D**). * *p* < 0.05 compared to HG, ^#^ *p* < 0.05 compared to HG + Con-Si (**G**–**I**).

To study whether Stat3 is the upstream of CAS in its regulation of hyperglycemia-induced vascular calcification, both HO-3867 (HO, a selective Stat3 inhibitor, 5 µM) and RNA interference were employed. As shown in Figure 6A, HO significantly reduced HG-stimulated Stat3 activation. Similar to NaHS, HO also obviously attenuated the downregulated elastin expression (Figure 6B) and upregulated CAS expression (Figure 6C) and activity (Figure 6D) caused by HG treatment. The effect of HO on HG-stimulated CAS expression was further validated by the immunohistochemical staining (Figure 6E). Following transfection of HASMCs with Stat3-specific siRNA, Stat3 expression was successfully suppressed (Figure 6F,G). Stat3 silencing also resulted in a significant decrease in CAS expression (Figure 6F,H) and an obvious increase in elastin level (Figure 6F,I).

### 2.7. Overexpression of WT-Stat3, but not that of Stat3 Mutant (C259S), Elevated CAS Protein Expression and Reduced Elastin Level in the Presence of HG

**Figure 7:**
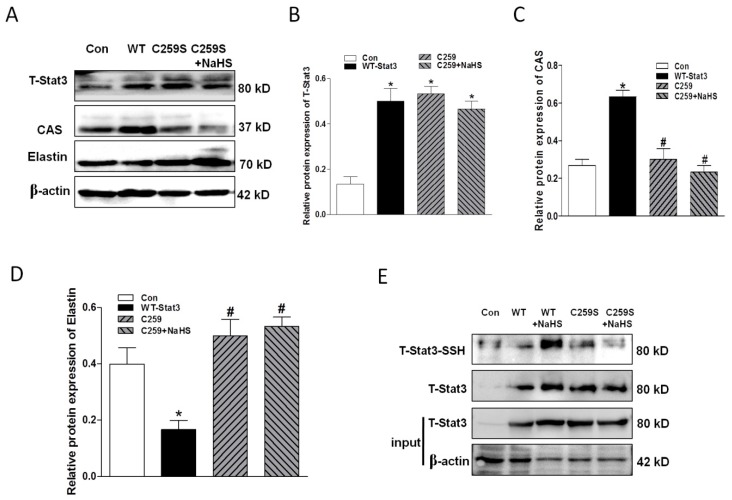
C259 of Stat3 is one of the targets of NaHS (**A**–**D**). Western blotting analysis showing that NaHS failed to produce effects on the protein expression of Stat3 (**A**,**B**), CAS (**A**,**C**) and elastin (**A**,**D**) in cells with overexpressed Stat3 mutant C259S. Experiments were performed in HASMCs cells treated with HG for 3 days. *n* = 3–5. The S-sulfhydration assay showing that NaHS induced S-sulfhydation only in WT-Stat3, but not in C259S mutant (**E**). * *p* < 0.05 compared to Control (Con), ^#^ *p* < 0.05 compared to WT-Stat3.

Dimerization of Stat3 is necessary for its activation and function. Stat3 dimerization can be mediated by interchain disulfide bridging involving Cys259 [34,35,36]. For this reason, we successfully transfected WT- and C259S Stat3 into HASMCs under condition of HG (Figure 7A,B). It was found that overexpression of WT-Stat3, but not C259S, elevated CAS protein expression and reduced elastin levels (Figure 7C,D). These were reversed upon transfection with Stat3 (C259S), NaHS application had no obvious effects on CAS expression and elastin levels. The *S*-sulfhydration assay with the tag-switch technique revealed that H_2_S induced *S*-sulfhydration in WT-Stat3 at the cysteine residue (Cys259), but not in the C259S mutant. This suggests that the S-sulfhydration at Cys259 may regulate Cys259 directly and then impair its dimer formation and activation (Figure 7E).

## 3. Discussion

In the present studies, we confirmed that high glucose promoted osteogenic transition and calcification of HASMCs. Moreover, we found that H_2_S administration not only inhibited the phenotype switching, but also ameliorated calcification of HASMCs. This was, at least partly, mediated by the up-regulation of the elastin level in the aorta through the inhibition of Stat3/CAS signaling.

We found in the present study that the calcification development of HASMCs was associated with the downregulated endogenous CSE/H_2_S in the smooth muscle cells. Pharmacological inhibition of CSE activity aggravated calcification, stimulated Stat3 activation, increased CAS activity, and reduced the elastin expression in HASMCs cultured by HG. Consistently, HG-induced activation of Stat3, an increase of CAS activity, and a reduction of the elastin level were significantly attenuated by CSE overexpression. These data suggest that endogenous H_2_S may be an important player to prevent calcification development in diabetes.

Stat3, an inducible monomeric transcription factor, dimerizes upon phosphorylation, and then translocates to the nucleus for exerting its functions on cell proliferation, angiogenesis, apoptotic resistance, and tumor evasion [37,38]. Stat3 also regulates inflammation and immune responses in vascular dysfunction [22,23]. High glucose induces ER stress and inflammation through the activation of Stat3 in diabetic retinopathy [25]. It has been put forward that Stat3 activation can induce the production of the cytokine transforming growth factor-β1 (TGF-β1), and it responds to intracellular ROS, and oxidative stress that may be a major contributor to diabetes [35]. Since ER stress, inflammation, TGF-β1, and oxidative stress are the risk factors for VC under a hyperglycemic state, it is reasonable to speculate that the inhibition of Stat3 signaling may be an effective strategy to inhibit calcification. In this study, we found that Stat3 was activated in HASMCs treated by HG, and the effect was restrained by H_2_S administration. Suppression of Stat3 phosphorylation with a Stat3 inhibitor and Stat3 silencing produced a similar effect. These results confirm that Stat3 signaling may be involved in the development of calcification caused by hyperglycemia. Stat3 dimerization is also mediated by interchain disulfide bridging involving Cys259 [35,36]. One of the most important mechanisms associated with the physiological effects of H_2_S in various body systems is post-translation modification, which controls the activity of many proteins. H_2_S can react with particular cysteine residues through converting cysteine –SH (thiol) groups to –SSH (persulfide) groups [39,40]. To confirm this, we detected S-sulfhydration in both WT and C259S-Stat3. Our results showed that the mutant Stat3 (C259S) downregulated CAS expression. The data imply that Cys259 plays an important role in the regulation of the expression of CAS. Interestingly, H_2_S had no significant effect on calcification in cells overexpressing C259S-Stat3. H_2_S may act on and modify the molecular structure of C259 to attenuate Stat3 activation. The S-sulfhydration assay further confirmed that H_2_S can induce S-sulfhydration at the cysteine residue (Cys259). This sulfhydration may impair the dimer formation of Stat3 and result in the anti-calcific effects in HASMCs-treated by HG.

CAS, a cysteine protease, can degrade the basement membrane and surrounding extracellular matrix of arterial walls. It has been reported that CAS is implicated in mediating elastin degradation and calcification in previous studies of atherosclerotic arteries of uremic, ApoE^−/−^ mice [41], and earlier Sukhova et al. demonstrated that the reduced atherosclerosis was found in CAS-deficient, LDL-receptor-deficient mice [30]. Elastin is an important component for consisting of elastic fibers, and it is synthesized and secreted from medial VSMCs for the maintenance of the VSMC phenotype and the vascular environment [26,27]. Moreover, elastin loss and disruption of elastin fibers were associated with aortic stiffening and remodeling in CKD [30] or STZ–diabetic rats [42]. Consequently, inhibition of CAS to improve elastin levels could be a useful therapy for preventing VC. In the present study, H_2_S inhibited the increases of CAS expression and activity in vivo and in vitro, and that those were significantly inhibited by the Stat3 inhibitor. Moreover, down-regulation of Stat3 also inhibited CAS expression in vitro. Interestingly, we observed that elastin was reduced markedly in HG-cultured HASMCs, and they were improved by H_2_S administration or the pharmacological inhibition of Stat3 and CAS, respectively. Moreover, the overexpression of CAS caused the further decrease in elastin and promoted the change of osteogenic phenotype in HASMCs. Collectively, these observations demonstrated that Stat3/CAS mediates calcification by affecting elastin degradation, and H_2_S inhibiting calcification is partly associated with the Stat3/CAS/elastin pathway.

The pathophysiological significance of H_2_S has been extensively studied in numerous diseases such as cerebral, cardiovascular, and renal diseases [43,44,45,46]. Emerging evidence has suggested that H_2_S also actively regulates vascular function and is implicated in vascular diseases including vascular calcification [47]. In the recent decade years, remarkable progress has been made in the research of the therapeutic potential of H_2_S, and continued innovation of its synthetic donors drives H_2_S research forward [48]. H_2_S donors such as the hydrolysis-triggered donor GYY4137 and so on [48], enable biological studies on the pathophysiological roles of H_2_S in vascular calcification. Although many important questions remain unanswered in the field of H_2_S donors, persistent innovation in synthetic donors and increased understanding of H_2_S physiology may eventually enable a pathway to the clinic for vascular calcification.

The present study demonstrated that H_2_S offers protection against calcification by mitigating Stat3/CAS signaling cascades for increasing local elastin levels. Further studies are needed to determine the precise mechanism by which H_2_S affects Stat3/CAS signaling involving the inhibition of VC in diabetic patients.

## 4. Materials and Methods

### 4.1. Reagents

Tris, glycine, NaCl, SDS, mannitol, bovine serum albumin (BSA), sodium hydrosulfide (NaHS), β-glycerophosphate (β-GP), ascorbate, and propargylglycine (PAG) were purchased from Sigma-Aldrich (St Louis, MO, USA). HO-3867 and LY3000328 (LY) were from Medchem express, LLC (Princeton, NJ, USA). Dulbecco’s modified Eagle’s medium (DMEM), fetal bovineserum (FBS), streptomycin/penicillin and trypsin were obtained from Hyclone Laboratories (South Logan, UT, USA). The RIPA buffer was purchased from Thermo Fisher Scientific Inc (Waltham, MA, USA). The Bradford colorimetric protein assay kit (Rockford, IL, USA) was used for protein quantification. The calcium, phosphorus, and alkaline phosphatase (ALP) kits were purchased from Jiancheng Bioengineering Co (Nanjing, China).

### 4.2. Cell Culture

HASMCs (Cell Resource Center, SIBS, CAS) were cultured according to the manufacturer’s instructions. HASMCs were incubated in Dulbecco’s modified Eagle medium (GE Healthcare HyClone Cell Culture Media) and 10% HyClone fetal bovine serum (GE Healthcare Bio-Sciences, Pasching, Austria). The calcifying medium contained 10 mM β-GP and 50 μg/mL ascorbate. Two concentrations of glucose (1000 mg/L or 4500 mg/L) were used. Osmolarity in the normal glucose was corrected by addition of mannitol (3500 mg/L) into the normal glucose (1000 mg/L) medium, and the medium was changed every 2 days.

### 4.3. Determination of Calcification

Mineralization of HASMCs was induced using high glucose (4500 mg/L) under a Calcifying medium containing β-GP and ascorbate for 14 days. Cells were fixed with 4% formaldehyde for 10–15 min and washed out, followed by an incubation with alizarin red (1%, *w*/*v*, pH 4.2) for 5 min, and distilled water was used for a final rinse for half an hour. Positive calcium staining is red/orange.

### 4.4. Measurement of Calcium Content

O-cresolphthalein colorimetric (OCPC) method was used for calcium content measurement of the HASMCs, which were decalcified with HCl at 37 °C for 24 h. The mixed working reagent solution containing ethanolamine buffer, OCPC, and 8-hydroxyquinoline were added into the supernatant fluid. The solutions were incubated at 30 °C for 5 min. The absorbance of the compound solution was measured at 600 nm.

### 4.5. Measurement of Phosphorus Level

Phosphomolybdic acid method was used for phosphorus content examination. The precipitating agent was added into the cell lysate. After that, the mixed solution was centrifuged at 3500 r/min for 10 min, and working solution containing hosphomolybdic acid was added into the supernatant fluid. The compound solution was incubated at 37 °C for 30 min, and the absorbance was measured at 660 nm.

### 4.6. Measurement of Alkaline Phosphatase (ALP)

Proteins were extracted from the HASMCs lysate in 0.05% Triton X-100 in PBS, respectively. Total proteins were quantified using a bicinchoninic acid (BCA, ThermoFisher, Waltham, MA, USA) protein assay. The supernatant was mixed with reaction mixture, respectively. The mixed solution was incubated at 37 °C for 15 min, then developer was added into each well, and the absorbance was determined at 520 nm. ALP activity was calculated according to the manufacturer’s instructions.

### 4.7. Measurement of H_2_S Synthesis Enzymes Cystathionine-γ-lyase (CSE) Activity

The H_2_S production rate in aorta or HASMCs was measured as described previously [15]. Briefly, HASMCs were harvested and rat aortic tissues from each group were homogenized in 100 mM potassium phosphate buffer (pH 7.4). The assay was performed in the present of 10 mM L-cysteine, and 2 mM pyridoxal 5′-phosphate. After incubation at 37 °C for 30 min, zinc acetate (1% *w*/*v*) was added to trap H_2_S, trichloroacetic acid (10% *w*/*v*) was used to stop the reaction. Equal volumes of *N*,*N*-dimethyl-p-phenylenediamine sulfate (20 mM) and FeCl_3_ (30 mM) were next added and centrifuged for 10 min, the absorbance of supernatant at 670 nM was determined.

### 4.8. Measurement of CAS Activity

After measurement of the protein concentration, the homogenate of cell lysate (100 µg) was incubated in chilled buffer on ice for 10 min and then centrifuged at a top speed in a microcentrifuge for 5 min, respectively. The supernatant was transferred to a new tube. 50 µL of supernatant, 50 µL of reaction buffer, and 2 µL of the 10 mM substrate were added orderly into the 96-well plate. After incubation at 37 °C for 2 h, samples were measured by a Varioskan Flash microplate reader from Thermo Electron Corporation (Waltham, Mass, USA) at the wavelength of 400 nm excitation and 505 nm emission.

### 4.9. Western Blot Analysis

Protein extracted from the HASMCs lysate was prepared and analyzed by Western blotting. Homogenate made from the harvested HASMCs was lysed with a RIPA buffer containing phosphatase and protease inhibitor. The protein quantification was measured using BCA method. Equal amounts of protein (40 μg) were separated by 10% SDS-polyacrylamide gel electrophoresis (SDS/PAGE) and proteins were transferred to nitrocellulose membrane (Bio-Rad). After blocking with 10% nonfat milk, the membranes were probed with primary antibodies against SM22α, SMα-actin, elastin, CSE (Santa Cruz Biotechnology, Santa Cruz, CA, USA), P-Stat3, T-Stat3, Cbfα-1 (Cell Signaling Technology, 3 Trask Lane Danvers, MA, USA) or CAS (Abcam, Cambridge, MA, USA) overnight at 4 °C. Horseradish peroxidase–conjugated anti-mouse, anti-rabbit or anti-sheep IgG were used as a secondary antibody. The immunoblots were visualized with an Enhanced Chemiluminescence Kit (ECL™ Plus, GE Healthcare, Piscataway, NJ, USA) and immunodetection was performed using and autoradiography. Protein band intensity was normalized with non-phosphorylated Stat3 or β-actin levels.

### 4.10. Immunofluorescence

HASMCs were fixed with acetone/methanol (1:1) for 10 min. After permeabilization with 0.1% Triton x-100 in PBS for 10 min at room temperature, cells were blocked in bovine serum albumin (BSA)/PBS for 1 h, then incubated with primary antibody (CAS, 1:50, ab18822) diluted in blocking buffer, at 4 °C overnight. Secondary antibody CY3-conjugated anti-sheep IgG (red) was used for fluorescence detection (1:200, Molecular Probes, Thermo Fisher Scientific, Waltham, MA, USA). Nuclei were counterstained with DAPI.

### 4.11. Transfection of HASMCs with CAS and Stat3 Plasmid, and Stat3 siRNA

The construct of CAS was kindly provided by Dr. Hyun-Shik from School of Life Science and Biotechnology, College of Natural Sciences, Kyungpook National University, Republic of Korea. Human STAT3 small interfering RNA (sc-29493; Santa Cruz Biotechnology, Santa Cruz, CA, USA). Wild type plasmid Stat3 (WT-Stat3) site directed mutagenesis was performed with the Phusion Site-Directed Mutagenesis Kit from Thermo Fisher Scientific Inc based on an overlap extension polymerase chain reaction with pXJ40-Stat3 as template. The PCR products (WT-Stat3 and Stat3 C259S) were inserted into a pIRESPuro3 vector. The plasmids were confirmed by DNA sequencing. Stat3 C259S plasmid was transfected in HASMCs by using an Invitrogen Lipofectamine 3000 transfection kit (Carlsbad, CA. USA). After transfection for 24 h, cells were treated with different chemicals. The transfection efficiency was determined by Western blots.

### 4.12. S-sulfhydration Assay

Stat3 S-sulfhydration was determined with the tag-switch technique [34]. Briefly, cells transfected with flag-tagged Stat3 or Mu-Stat3 (C259S) were treated with or without 100 µM NaHS for 30 min, then cells were collected and resuspended in HEN buffer (250 mM HEPES, 50 mM NaCl, 1 mM Ethylene Diamine Tetraacetic Acid (EDTA), 0.1 mM neocuproine, 1% NP-40) containing protease inhibitors. After ultrasonication, 50 µL of the cell lysates was mixed with 50 mM MSBT-A and incubated in water batch at 37 °C for 1 h. Subsequently, the mixture was desalted and pulled down with protein A/G beads by anti-flag antibody. After washing for 3 times by using PBS with protease inhibitors, the beads were incubated with 20 mM biotin-linked cyanoacetate in PBS containing 2.5% SDS at 37 °C for 1 h. The excessive biotin-linked cyanoacetate in the supernatant was removed after centrifuge. The remaining beads were mixed with 30 µL non-reducing loading buffer and boiled at 95 °C for 1 min. Western blot method was used to analyze the resulted samples. S-sulfhydration of Stat3 was tested with anti-biotin antibody, and the total Stat3 protein was detected with anti-flag antibody after stripping.

### 4.13. Statistical Analysis

Data are expressed as the mean ± SEM. One-way or two-way ANOVA followed by Bonferroni’s post-hoc test was used analyze multiple comparisons. *P* < 0.05 was considered statistically significant.
